# Improvements in Adolescents’ Disordered Eating Behaviors in a Collaborative Care Digital Mental Health Intervention: Retrospective Observational Study

**DOI:** 10.2196/54253

**Published:** 2024-01-31

**Authors:** Landry Goodgame Huffman, Darian Lawrence-Sidebottom, Aislinn Brenna Beam, Amit Parikh, Rachael Guerra, Monika Roots, Jennifer Huberty

**Affiliations:** 1 Bend Health Inc Madison, WI United States; 2 FitMinded Inc LLC Phoenix, AZ United States

**Keywords:** behavioral care, mental health, web-based coaching, web-based therapy, eating disorders, eating, anorexia, coach, coaching, pediatric, pediatrics, adolescent, adolescents, teen, teens, teenager, teenagers, digital mental health intervention, DMHI, collaborative, digital health

## Abstract

**Background:**

Young people today are exhibiting increasing rates of disordered eating behaviors, as well as eating disorders (EDs), alongside other mental and behavioral problems such as anxiety and depression. However, limited access to mental health care means that EDs, disordered eating behaviors, and comorbid mental health problems are often underdiagnosed and undertreated. Digital mental health interventions (DMHIs) offer accessible and scalable alternatives to traditional treatment modalities, but their effectiveness has not been well established among adolescents with EDs and disordered eating behaviors.

**Objective:**

This study uses data from a collaborative care pediatric DMHI to determine whether participation in a DMHI is associated with a reduction in adolescents’ disordered eating behaviors.

**Methods:**

Adolescent members in care with Bend Health Inc completed the SCOFF questionnaire at baseline (before the start of care) and approximately every month during care to assess disordered eating behaviors. They also completed assessments of mental health symptoms at baseline. Member characteristics, mental health symptoms, and disordered eating behaviors of adolescents with elevated SCOFF scores at baseline (before the start of care) were compared to those of adolescents with nonelevated SCOFF scores at baseline. Members participated in web-based coaching or therapy sessions throughout the duration of mental health care.

**Results:**

Compared to adolescents with nonelevated SCOFF scores (n=520), adolescents with elevated SCOFF scores (n=169) were predominantly female and exhibited higher rates of elevated anxiety and depressive symptoms. SCOFF scores decreased over time in care with the DMHI for 61.4% (n=70) of adolescents with elevated SCOFF scores, and each additional month of participation was associated with greater improvements in disordered eating behaviors (*F*_1,233_=72.82; *P*<.001).

**Conclusions:**

Our findings offer promising preliminary evidence that participation in mental health care with a collaborative care DMHI may be beneficial in the reduction of disordered eating symptoms in adolescents, including those who are experiencing comorbid anxiety and depressive symptoms.

## Introduction

Eating disorders (EDs) and associated disordered eating behaviors impact approximately 10% of the US population [1]. The peak age of onset of EDs such as anorexia nervosa, bulimia nervosa, avoidant or restrictive food intake disorder, and binge ED is between 13 and 18 years, making them particularly relevant during adolescence. Estimates of EDs in adolescence range from 1.2% among males to 5.7% in females and 9% among sexual and gender nonconforming youths [2-5]. Disordered eating behaviors (eg, obsessing over food intake and excessive worry about weight) are even more common, impacting nearly 1 in 4 children and adolescents [6]. EDs and disordered eating behaviors disrupt critical periods of physical and socioemotional development that occur during childhood and adolescence [7]. Youths with EDs experience compromised physical functioning such as malnutrition, disrupted pubertal development, and delayed menarche, as well as worsened psychosocial functioning. EDs are highly comorbid with, and often preceded by, other mental health problems such as anxiety or anxiety disorders, depression, and obsessive-compulsive disorder [8].

During the COVID-19 pandemic, the prevalence of EDs more than doubled among adolescents, exacerbating an already pressing public health issue [9,10]. Indeed, EDs confer a significant economic burden for families and hospitals, with an estimated annual disease burden of US $70 billion [11,12]. However, more than 75% of those with EDs or risk for EDs do not receive the necessary treatment [13]. Several issues underlie this gap between ED diagnosis and treatment. ED treatments are often difficult to access, especially for young people, who often experience financial, geographic, and transportation constraints as well as increased stigma [14]. Additionally, in-person services, such as inpatient and outpatient care and face-to-face therapy, are severely limited in their accessibility, largely due to shortages of trained personnel and long waitlists [14].

In recent years, digital mental health interventions (DMHIs) such as self-guided applications and web-based therapy have emerged as accessible and scalable alternatives to traditional mental health treatments. Several systematic reviews and meta-analyses suggest the potential for DMHIs in ED treatment, although results remain heterogeneous and inconclusive [15]. Moreover, few studies of DMHIs for EDs have been conducted among adolescents, despite the pressing need for accessible child and adolescent ED treatments. As argued by Loucas et al [16], most pediatric DMHIs for EDs are more similar to web-based self-help programs than therapeutic interventions, given their lack of personalized and interactive components [17-19]. However, recent advances in pediatric DMHIs using family-based therapy for ED treatment have yielded promising results, suggesting that digital ED treatments for youths are most effective when administered in the context of a holistic care team [20-22].

The collaborative care model is a team-based framework for mental health care that has been used by DMHIs with promising results. In the collaborative care model, primary care providers collaborate with behavioral care managers (BCM) and other providers to implement measurement-based mental health care. Collaboration between providers reduces the burden on primary care providers while ensuring optimal, evidence-based care via regular symptom assessments. As a result, interventions that use collaborative care models are particularly effective for the treatment of mental health problems in both youths and adults [23-26]. However, no studies to date have evaluated whether participation in collaborative care DMHIs may improve disordered eating behaviors among adolescents. As such, the purpose of this study was to use retrospective analyses of data collected from adolescents participating in care with a collaborative care DMHI to determine whether participation in a pediatric collaborative care DMHI is associated with a reduction in disordered eating behaviors. Considering established demographic and clinical correlates of disordered eating behaviors we also explored associations between potential confounds (age, sex, and anxiety and depressive symptoms) and disordered eating behaviors among those receiving care with the DMHI.

## Methods

### Design and Participants

All adolescents (ages 13-17 years) who met the following inclusion criteria were eligible for inclusion in the study (N=689): (1) started mental health care (first synchronous event) with Bend Health Inc between January 1, 2023, and October 1, 2023 (9 months); (2) had at least 1 synchronous session with a Bend Health Inc coach or therapist during the study time frame; and (3) completed the assessment of eating behaviors before the start of care (baseline).

### Treatment

Bend Health Inc is a DMHI that provides behavioral care for adolescents (aged 13-17 years), using a whole-family approach (ie, caregivers are closely involved in care), via a web-based platform. Bend Health Inc’s behavioral care has been described elsewhere [24,25]. Members enroll via referral from a health care provider, or they use insurance, employer benefits, or self-pay. Members are assigned a BCM, who conducts an initial evaluation of the member’s mental health concerns and circumstances, and they continue to monitor the member’s care while they are enrolled in the program. BCMs assign members a coach, and also a therapist in some cases, based on their mental health symptoms (eg, type and acuity), goals for treatment, and insurance coverage; therapists tend to be assigned only to members with more severe symptoms and conditions, whereas nearly all members are assigned a coach. Members with higher symptom acuity or a psychiatric referral may also be assigned a prescribing psychiatric practitioner (eg, a medical doctor or psychiatric nurse practitioner). In synchronous video-based coaching and therapy sessions, Bend Health Inc’s practitioners guide members and their caregivers through structured care programs. These care programs are designed to deliver evidence-based tools and techniques that target common mental and behavioral health issues such as anxiety, depression, and body image. Most care programs are designed to be completed in approximately 3 months, and some care programs (eg, the body image program) are designed to be completed in a shorter amount of time. Once a month, caregivers and adolescent members are asked to complete validated web-based assessments of the adolescent’s mental health symptoms (see Measures section). Caregivers are required to be in the same general location for their adolescents’ synchronous sessions with a coach or therapist for safety purposes.

### Measures

Demographic and health information of adolescent participants is gathered during enrollment with Bend Health Inc. Caregivers respond to basic demographic questions, providing their adolescent member’s date of birth, sex at birth (male, female, or others), gender identity (male, female, transgender, nonbinary, or others), and race or ethnicity. Details on the race or ethnicity response options are included in Multimedia Appendix 1.

In addition, at enrollment, caregivers and adolescent members complete a series of symptom screening questions and validated assessments to identify common mental and behavioral health concerns. To assess eating behaviors, all adolescent members complete the SCOFF (Cronbach α=.48) [27,28]. The SCOFF is a validated questionnaire, in which the member responds “yes” or “no” to 5 items about disordered eating behaviors that they might have. The 5 items, which each correspond with a letter of the SCOFF name, are as follows: (1) Do you make yourself *s* *ick* because you feel uncomfortably full? (2) Do you worry that you have lost *c* *ontrol* over how much you eat? (3) Have you recently lost more than *o* *ne* stone (14 lb) in a 3-month period? (4) Do you believe yourself to be *f* *at* when others say you are too thin? and (5) Would you say that *f* *ood* dominates your life?

To identify members with elevated anxiety and depressive symptoms, caregivers of adolescent members respond to 5 screening questions drawn from the *Diagnostic and Statistical Manual of Mental Disorder, Fifth Edition, Text Revision* (DSM-5-TR) Cross-Cutting Symptom Measure [29]. For anxiety, the caregivers and adolescents together respond to the following questions: *Over the last 2 weeks, how often have you been bothered by any of the following problems? (1) Feeling nervous, anxious, or on edge? and (2) Not being able to stop or control worrying?* For depressive symptoms, caregivers and teens responded to the items: *Over the last 2 weeks, how often have you been bothered by any of the following problems? (1) Had less fun doing things than you used to? and (2) Felt sad or depressed for several hours?* Best-fit responses are selected from a 4-item Likert-type scale, with responses ranging from “not at all” (item score=0) to “nearly every day” (item score=3). Screener scores are calculated by aggregating all item scores for each screener.

Members who have an anxiety screener score of 2 or more are prompted to complete the Generalized Anxiety Disorder-7 (GAD-7) assessment (Cronbach α=.91) [30,31], and members who have a depressive screener score of 2 or more are prompted to take the Patient Health Questionnaire-9 for Adolescents (PHQ-9A; Cronbach α=.85) [32,33]. The GAD-7 has 7 questions regarding symptoms of anxiety in the prior 2 weeks with the same Likert-type scale as used in the screener questions (“not at all” to “nearly every day”). The PHQ-9A is a modified version of the PHQ-9 for adolescents aged 11-17 years. The original measure has 9 questions, but we omit a question regarding suicide and self-harm (ie, the PHQ-9A here includes 8 questions). The PHQ-9A asks adolescents about depressive symptoms in the prior week using the same Likert-type scale as in the GAD-7 and screener questions. Adolescents are asked to report their own symptoms for both GAD-7 and PHQ-9A.

### Statistical Analysis

#### Outcome Calculations

The last assessment before the start of care (baseline) and all assessments after the start of care were considered for analysis. SCOFF scores were calculated by aggregating the number of “yes” responses, with scores ranging from 0 to 5. As has been used previously [27], members with a SCOFF score of 2 or more on their baseline assessment (ie, the last assessment before the start of care) were included in the “elevated SCOFF score” group, and members with a score of less than 2 were included in the “nonelevated SCOFF score group.” GAD-7 scores were calculated by aggregating the individual item scores. PHQ-9A scores were calculated by aggregating the individual item scores, and then, dividing by 8 and multiplying by 9 (to account for the omitted item). Members with moderate or greater severity anxiety or depressive symptoms, as determined by established criteria for the GAD-7 and PHQ-9A [29,30], were flagged as having elevated anxiety or depressive symptoms, respectively.

#### Baseline Characteristics: Eating Behaviors and Member Characteristics

SCOFF scores at baseline were characterized for all members, including total score and responses to individual SCOFF items. Then, member characteristics, anxiety and depressive symptom severity (elevated or nonelevated), and care participation characteristics were reported for each group and compared between groups to identify any differences. Member characteristics included age at baseline, sex (female, male, and nonbinary), gender-sex conformity (conforming and nonconforming), race or ethnicity (Asian, Black or African American, Hispanic or Latino, White, and other or multiracial), mental health condition (anxiety disorder diagnosis and depressive disorder diagnosis), and elevated mental health symptoms (anxiety and depression). The care participation metrics included the number of months in care (time between first session and last session), rates of members in coaching and therapy, and rates of members participating in the anxiety, depression, and body image care programs. Member demographics were reported by caregivers (described earlier), mental health conditions were identified from electronic health records, mental health symptoms were characterized based on symptom severity at baseline, and care participation characteristics were assessed using data from electronic health records. Age in years and months in care were compared between groups using Wilcoxon signed rank tests. All other between-group comparisons for member characteristics were performed using chi-square tests.

#### Change in Eating Behaviors

The number of total SCOFF assessments (ie, with baseline as the first assessment) was quantified for members in both groups. Only data from members with at least 2 assessments (baseline and at least 1 assessment after care) were included in the analyses of change in SCOFF scores (n=233 members excluded). For members in both groups, SCOFF scores at baseline and the last assessment, as well as the change in score from baseline to last assessment, were quantified. Change scores were compared to 0 using Wilcoxon signed rank test to determine whether SCOFF scores changed significantly over the course of care. The rates of members with a decrease and an increase in SCOFF score were reported for both groups to quantify rates of symptom improvement and symptom worsening, respectively. To identify which items contributed to a change in SCOFF score, “yes” responses to each item at the last assessment were reported for members who responded “yes” to the item at baseline.

Finally, a linear mixed effects model was used to test whether SCOFF scores decreased over months in care and to test whether mental health symptom severity (at baseline) and demographic factors predicted SCOFF scores. Only members with a baseline assessment within 1 month or less of the start of care were included in the linear mixed effects model (n=10 members excluded). The basic model included a fixed effect of months in care (at the time of SCOFF assessment) and a random effect of subject (member ID) on the intercept. Alternative models including one of the following predictors were tested for fit against the basic model using likelihood ratio tests: elevated anxiety symptoms at baseline (yes or no), elevated depressive symptoms at baseline (yes or no), sex at birth (female or nonfemale), age at baseline (in years), and participation in therapy (yes or no). When a likelihood ratio test was statistically significant, the predictor variable was included in the final model as a fixed effect.

Throughout, standard descriptive statistics (eg, percentages, mean and SD, and median and IQR) were used to describe the data, as appropriate. IQR values are reported as the range: 25th-75th percentile. An α level of .05 was used as the threshold of statistical significance for all analyses. Tests of normality (Kolmogorov-Smirnov and Jarque-Bera) were performed to determine appropriate statistical tests and descriptive statistics. *P* values were corrected for multiple tests using the Bonferroni Hoc correction statistical tests performed on baseline characteristics and change in SCOFF scores (2 sets of corrections).

### Ethical Considerations

At enrollment with Bend Health Inc, all study participants provided informed consent for primary data collection (required for participation in care) and use of their data in further analyses. Given the retrospective observational nature of the study, participants were not compensated for their participation in the study. Procedures for this study were approved by the Biomedical Research Alliance of New York (Study 23-12-034-1374; approved on June 5, 2023). To ensure the privacy and confidentiality of the human participants in this study, all data (eg, from electronic health records) were deidentified prior to analysis.

## Results

### Baseline Characteristics: Eating Behaviors and Member Characteristics

The distribution of baseline SCOFF scores is reported in Table 1. Ultimately, 75.5% (n=520) had nonelevated SCOFF scores, and 24.5% (n=169) had elevated SCOFF scores. For members with nonelevated SCOFF scores, 13.7% (n=71) of members responded “yes” to the item about control (item 2), and 9.2% (n=48) responded “yes” to the item about believing you are fat (item 4; Table 2). For members with elevated SCOFF scores, the most commonly reported items were loss of control (item 2: n=151, 89.3%), believing you are fat (item 4: n=113, 66.9%), and food dominating life (item 5: n=92, 54.4%).

Compared to the nonelevated SCOFF score group, the elevated SCOFF score group was more predominantly female (*χ*^2^_1_=24.2; *P*<.001) and also had a higher rate of diagnoses with depressive disorders (*χ*^2^_1_=9.5; *P*=.005; Table 3). Rates of elevated anxiety and depressive symptoms were higher for members with elevated SCOFF scores than members with nonelevated SCOFF scores (anxiety: *χ*^2^_1_=31.9; *P*<.001 and depression: *χ*^2^_1_=63.2; *P*<.001). Age, gender-sex conformity, race or ethnicity, and rates of anxiety disorder diagnoses did not differ between groups.

Members were in care for a median of 2.60 (IQR 1.27-4.23) months. In terms of participation in behavioral care, 98.8% (n=681) of all members were in coaching, and 39.8% (n=274) of all members were in therapy. Rates of participation in coaching and therapy, as well as months in care, did not differ between groups. Approximately 1 in 2 members participated in the anxiety care program, and this was similar between groups. However, participation in the depression and body image care programs was higher for members with elevated SCOFF scores versus members with nonelevated SCOFF scores (depression: *χ*^2^_1_=15.0; *P*<.001).

**Table 1:** Distribution of SCOFF scores at baseline (N=689).

SCOFF score	Members, n (%)
0	368 (53.4)
1	152 (22.1)
2	100 (14.5)
3	50 (7.3)
4	13 (1.9)
5	6 (0.9)

**Table 2:** Responses to each SCOFF item at baseline by group.

SCOFF item	Nonelevated SCOFF score (n=520), n (%)	Elevated SCOFF score (n=169), n (%)
Do you make yourself *sick* because you feel uncomfortably full?	13 (2.5)	43 (25.4)
Do you worry that you have lost *c* *ontrol* over how much you eat?	71 (13.7)	151 (89.3)
Have you recently lost more than *o* *ne* stone (14 lb) in a 3-month period?	11 (2.1)	33 (19.5)
Do you believe yourself to be *f* *at* when others say you are too thin?	48 (9.2)	113 (66.9)
Would you say that *f* *ood* dominates your life?	9 (1.7)	92 (54.4)

**Table 3:** Member characteristics for each group.

Member characteristics			Nonelevated SCOFF score (n=520, 75.5%)		Elevated SCOFF score (n=169, 24.5%)		Comparison		
							Chi-square (*df*=1)		*P* value
Age (years), median (IQR)^a^			15 (14-16)		15 (14-16)		N/A^b^		.87
**Sex, n (%)**									
	Female	301 (57.9)		134 (79.3)		24.2		<.001	
	Male	212 (40.8)		33 (19.5)		N/A		N/A	
	Nonbinary	7 (1.3)		2 (1.2)		N/A		N/A	
**Gender-sex conformity, n (%)**									
	Conforming	481 (92.5)		155 (91.7)		0.03		.87	
	Nonconforming	39 (7.5)		14 (8.3)		N/A		N/A	
**Race or ethnicity, n (%)**									
	Asian	30 (5.8)		4 (2.4)		N/A		N/A	
	Black or African American	35 (6.7)		15 (8.9)		N/A		N/A	
	Hispanic or Latino	25 (4.8)		17 (10.1)		N/A		N/A	
	White	252 (48.5)		87 (51.5)		0.4		.66	
	Other or multiracial	178 (34.2)		46 (27.2)		N/A		N/A	
**Mental health condition, n (%)**									
	Anxiety disorder	147 (28.3)		56 (33.1)		1.2		.46	
	Depressive disorder	35 (6.7)		25 (14.8)		9.4		.005	
**Elevated mental health symptoms, n (%)**									
	Anxiety	230 (44.2)		118 (69.8)		31.9		<.001	
	Depression	211 (40.6)		129 (76.3)		63.2		<.001	
Duration in care (months), median (IQR)^c^			2.51 (1.23-4.20)		2.80 (1.50-4.37)		N/A		.28
**Behavioral care participation, n (%)**									
	Coaching	513 (98.7)		168 (99.4)		N/A		N/A	
	Therapy	201 (38.7)		73 (43.2)		0.92		.50	
**Care program, n (%)**									
	Anxiety	243 (46.7)		84 (49.7)		0.3		.66	
	Depression	77 (14.8)		48 (28.4)		15.0		<.001	
	Body image	6 (1.2)		7 (4.1)		N/A		N/A	

^a^Between-group comparison was performed using a Wilcoxon signed rank test (*z*=–0.23).

^b^N/A: not applicable.

^c^Between-group comparison was performed using a Wilcoxon signed rank test (*z*=–1.46).

### Change in Eating Behaviors

SCOFF assessment counts during the study time frame are reported for each group in Table 4. For all members with a baseline and postcare assessment (ie, 2 or more total assessments), baseline assessments were completed at a median of 0.27 (IQR 0.5-0.13) months before the start of care, and the last assessments were completed at a median of 2.23 (IQR 1.1-3.6) months after the start of care. The timing of baseline and last assessments did not differ between groups (baseline: *z*=0.70; *P*=.70 and last assessment: *z*=–0.38; *P*=.70).

For members in the nonelevated SCOFF score group, 16.4% (n=56) had a decrease in SCOFF score from baseline to their last assessment, 13.7% (n=47) had an increase in SCOFF score, and 69.9% (n=239) had no change (Table 5). For members in the elevated SCOFF score group, on the other hand, 61.4% (n=70) had a decrease in SCOFF score from baseline to their last assessment, 16.7% (n=19) had an increase, and 21.1% (n=25) had no change. While SCOFF scores remained stable for the nonelevated SCOFF score group (*z*=–0.47; *P*=.70), median SCOFF scores decreased from 2 (IQR 2-3) at baseline to 1 (IQR 0-3) at the last assessment for the elevated SCOFF score group (*z*=–6.39; *P*<.001). Individual item responses at the last assessment for members who responded “yes” to the item at baseline are reported in Table 6.

**Figure 1:**
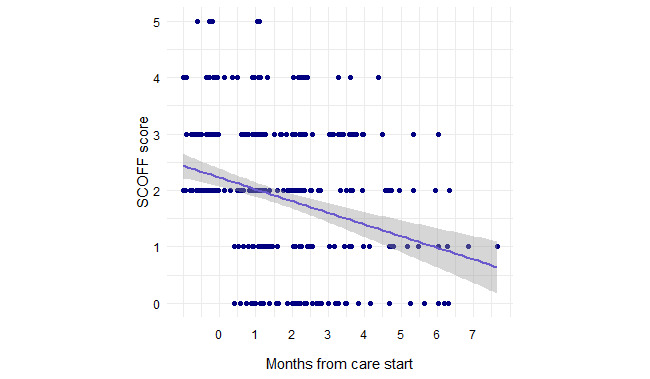
SCOFF score over months in care for members with elevated SCOFF scores. Individual scores are indicated by dark blue markers, and the linear model fit is indicated by the purple line.

In the linear mixed effects model of SCOFF score over time in care for members with elevated SCOFF scores, the following predictors were included in the final model: elevated anxiety symptoms at baseline (*χ*^2^_1_=7.6; *P*=.006), elevated depressive symptoms at baseline (*χ*^2^_1_=8.6; *P*=.003), and participation in therapy (*χ*^2^_1_=4.4; *P*=.03). SCOFF score decreased by 0.26 points for each month in care (*F*_1,233_=72.82; *P*<.001; Figure 1). Members with elevated anxiety symptoms at baseline had SCOFF scores 0.40 points higher than those with nonelevated anxiety symptoms (*F*_1,101_=9.87; *P=*.005). Similarly, members with elevated depressive symptoms at baseline had SCOFF scores 0.43 points higher than those with nonelevated depressive symptoms (*F*_1,101_=5.40; *P*=.044). Participation in therapy did not relate to SCOFF score (*F*_1,101_=2.87; *P*=.16).

**Table 4:** Total assessments completed for each group.

Total assessments	Nonelevated SCOFF score (n=520), n (%)	Elevated SCOFF score (n=169), n (%)
1	178 (34.2)	55 (32.5)
2	141 (27.1)	44 (26)
3	96 (18.5)	31 (18.3)
4	57 (11)	22 (13)
5	25 (4.8)	9 (5.3)
6	15 (2.9)	5 (3)
7+	8 (1.5)	3 (1.8)

**Table 5:** Change in SCOFF score from baseline to last assessment for each group.

		Nonelevated SCOFF score (n=342)	Elevated SCOFF score (n=114)
**SCOFF score, median (IQR)**			
	Baseline	0 (0 to 1)	2 (2 to 3)
	Last	0 (0 to 0)	1 (0 to 3)
	Change	0 (0 to 0)	–1 (–2 to 0)
**Change in score, n (%)**			
	Decrease	56 (16.4)	70 (61.4)
	Increase	47 (13.7)	19 (16.7)

**Table 6:** Responses to each SCOFF item at the last assessment for members who answered “yes” to the item at baseline. Results are reported for each group.

	Nonelevated SCOFF score (n=342), n/N (%)		Elevated SCOFF score (n=114), n/N (%)	
	“Yes”	“No”	“Yes”	“No”
Do you make yourself *s* *ick* because you feel uncomfortably full?	1/8 (12.5)	7/8 (87.5)	9/23 (39.1)	14/23 (60.9)
Do you worry that you have lost *c* *ontrol* over how much you eat?	21/44 (47.7)	23/44 (52.3)	49/102 (48)	53/102 (52)
Have you recently lost more than *o* *ne* stone (14 lb) in a 3-month period?	2/9 (22.2)	7/9 (77.8)	4/22 (18.2)	18/22 (81.8)
Do you believe yourself to be *f* *at* when others say you are too thin?	14/35 (40)	21/35 (60)	50/77 (64.9)	27/77 (35.1)
Would you say that *f* *ood* dominates your life?	1/6 (16.7)	5/6 (83.7)	34/60 (56.7)	26/34 (43.3)

## Discussion

### Principal Results

Using retrospective analyses of data collected from adolescents participating in care with Bend Health Inc, the purpose of this study was to determine whether mental health care with a collaborative care DMHI is associated with a reduction in disordered eating behaviors while also accounting for demographic and mental health symptom covariates. Disordered eating scores decreased throughout care with the DMHI for 61.4% (n=70) of adolescents with elevated disordered eating scores at baseline, and longer participation was associated with greater reductions in scores. Elevated disordered eating behaviors at baseline were associated with female sex and elevated mental health symptoms. This study provides preliminary evidence that mental health care with a DMHI may positively affect disordered eating behaviors.

Over the course of care with the DMHI, 61.4% (n=70) of adolescents with disordered eating behaviors exhibited fewer problematic eating behaviors throughout participation. Further, each additional month in care was associated with larger improvements in disordered eating behaviors, even while controlling for elevated mental health symptoms. These findings, which suggest the effectiveness of a pediatric DMHI in mitigating symptoms of disordered eating, are especially timely, as many youths with EDs and disordered eating symptoms are unable to access adequate care via in-person modalities—a lingering effect of the COVID-19 pandemic and current mental health crisis in youths [34,35]. Moreover, these results suggest that collaborative care DMHIs may mitigate disordered eating behaviors in a relatively short time frame, with the change in disordered eating behaviors assessed within this study sample after just 2.23 (IQR 1.1-3.6) months in care. Given the complex and chronic nature of disordered eating behaviors and EDs, it is understandable that health care professionals prefer team-based, integrated behavioral care (eg, the collaborative care model used by the DMHI in this study) for the identification and treatment of EDs [36-39].

Compared to those without disordered eating behaviors, adolescent members with disordered eating behaviors were more likely to be female than male. Extant literature suggests a similar trend, namely, that females tend to report more disordered eating behaviors and higher rates of ED diagnosis than their male peers [40,41]. However, this does not mean that disordered eating is not a problem among males. In this study, 19.5% (n=33) of adolescents flagged with problematic eating behaviors were male. Disordered eating behaviors may go underreported and unrecognized in males due to stigma [42], and ED diagnostic criteria may not accurately capture disordered eating behaviors that are more prevalent in males than females, such as preoccupation with gaining muscle mass and fear of losing weight [43,44]. Mental health providers and practitioners in both traditional and web-based modalities should continue to screen for disordered eating behaviors among all clients, regardless of sex and gender, while paying particular attention to muscle dysmorphia and excessive exercise [41].

We also found that adolescents with disordered eating behaviors also had higher rates of elevated anxiety and depressive symptoms than their peers who were not flagged with disordered eating behaviors. Moreover, having elevated anxiety and depressive symptoms at baseline was a significant predictor of more severe disordered eating behaviors among youths flagged with disordered eating behaviors. In children, adolescents, and adults, internalizing disorders such as anxiety and depression frequently co-occur with disordered eating behaviors and EDs [45-47]. This study sample exhibited higher rates of anxiety and depressive symptom comorbidity than previously reported. This is to be expected, given that all children and adolescents in this study were treatment-seeking, whereas previous estimates have included both treatment- and nontreatment-seeking individuals [45,46]. Notably, 95.9% (n=162) of members flagged with disordered eating behaviors participated in care programs other than the body image care program (namely, the anxiety and depression programs), and this group exhibited improvements in disordered eating behaviors, nonetheless. This finding adds to the body of literature suggesting that the overlap between disordered eating behaviors and internalizing problems such as anxiety and depression may have similar underlying constructs and thus may warrant similar treatment [45,48]. Further research is necessary to study the effectiveness of DMHIs in directly addressing disordered eating behaviors as well as treating comorbid EDs and internalizing problems among young people.

### Limitations and Future Directions

While our findings provide compelling evidence that participation in a collaborative care DMHI may be associated with improvements in disordered eating behaviors, there are several limitations. The SCOFF assessment has attracted criticism for lack of sensitivity and accuracy and other questionnaires. The Eating Attitudes Test [49], for instance, may indeed allow for more fine-grained assessment and diagnosis of eating behaviors. However, the SCOFF has been used and validated extensively as a screener and assessment of common EDs among adolescents [27,50,51]. Moreover, the SCOFF has a high correlation with other validated disordered eating surveys [52] and has also shown acceptable convergent validity when compared to clinical interviews [53].

It should be noted that the SCOFF questionnaire includes an item that may not be appropriate for adolescents. Adolescence is a time of puberty and rapid growth, and therefore, losing 14 pounds may indicate extreme weight loss and a significant health issue (more so than the other SCOFF items). Further, if an adolescent responds “yes” to this item at one time point, it is unlikely that they will respond “yes” in the next several months. Therefore, we investigated whether our findings were driven or altered by the inclusion of this potentially problematic item. We found that few participants in the study sample responded “yes” to this item (n=44, 7.3% at baseline), and follow-up analyses revealed that our longitudinal results did not substantively change when this item was removed from the calculation of SCOFF scores (Multimedia Appendix 2). Thus, the inclusion of this item did not confound the primary findings reported here.

Although we evaluated cross-sectional associations between improvements in disordered eating behaviors and internalizing problems at baseline, future research should explore longitudinal and bidirectional associations between disordered eating symptoms and changes in mental health symptoms among youths involved in collaborative care DMHIs. Extant research regarding causal associations between disordered eating behaviors and other psychiatric symptoms among those engaged in treatment has yielded heterogeneous results [54-56], but no relevant studies to date have been conducted among youths involved in a collaborative care DMHI.

### Concluding Remarks

Young people today are exhibiting increasing rates of disordered eating behaviors and EDs alongside other mental and behavioral problems such as anxiety and depression. Collaborative care DMHIs have the capacity to mitigate the growing mental health crisis by providing holistic and evidence-based care that is more accessible and scalable than traditional modalities. The findings from this study suggest that participation with a collaborative care DMHI such as Bend Health Inc may be beneficial in the reduction of disordered eating symptoms in adolescents. Future studies, particularly those bolstered by improved measurement of eating behaviors over time and with a larger and more diverse cohort of youths, are paramount to establishing the effectiveness of collaborative care DMHIs as an evidence-based provider of care for those with problematic eating behaviors and EDs.

## Appendix

Multimedia Appendix 1 Information on the race and ethnicity demographic response options, and also how these data were analyzed.

Multimedia Appendix 2 Additional analyses, and accompanying results, to determine SCOFF response patterns of participants. Follow-up analyses were performed with the response to item 3 excluded from calculations of total SCOFF score.

## Abbreviations

BCM: behavioral care manager
DMHI: digital mental health intervention
DSM-5-TR: Diagnostic and Statistical Manual of Mental Disorder, Fifth Edition, Text Revision
ED: eating disorder
GAD-7: Generalized Anxiety Disorder-7
PHQ-9A: Patient Health Questionnaire-9 for Adolescents

## Data Availability

The data sets generated and analyzed during this study are not publicly available because this would violate Bend Health Inc’s privacy. However, aggregated and anonymized data are available from the corresponding author on reasonable request.
