# Supplementary material for: Improvements in Adolescents’ Disordered Eating Behaviors in a Collaborative Care Digital Mental Health Intervention: Retrospective Observational Study
Source: JMIR Form Res. 2024 Jan 31;8:e54253. doi: 10.2196/54253 (PMC10867747; doi:10.2196/54253)
Supplement: Multimedia Appendix 1 [file formative_v8i1e54253_app1.docx]

**Multimedia appendix 1**

The race/ethnicity response options were changed part-way through the study to better capture member diversity, and to align data with U.S. census standards. Specifically, from January 2023 to May 25, 2023, the race/ethnicity options were: “American Indian or Alaska Native”, “Asian”, “Black or African American”, “Hispanic or Latino”, “Native Hawaiian or other Pacific Islander”, “White”, and “Other”. Starting May 26, 2023, the following response options were added: “Chinese”, “Vietnamese”, “Native Hawaiian”, “Filipino”, “Korean”, “Japanese”, “Chamorro”, “Other Asian”, “Other Pacific Islander”, “Some other race or multi-racial”, “Mexican, Mexican Am., Chicano”, “Puerto Rican”, “Cuban”. Ultimately race/ethnicity was described in the present paper using the categories: White, other or multi-racial, Black/African American, Hispanic/Latino, and Asian. Members with a reported race/ethnicity of “Chinese”, “Vietnamese”, “Filipino”, “Korean”, “Japanese”, “Chamorro”, “Other Asian”, or “Other Pacific Islander” were categorized as “Asian”. Members with a reported race/ethnicity of “Mexican, Mexican Am., Chicano”, “Puerto Rican”, “Cuban”, “Another Hispanic, Latino, or Spanish origin” were categorized as “Hispanic or Latino”. Members with a reported race/ethnicity of “Other” and those that selected multiple race/ethnicity options, were categorized as “Other or multi-racial”.
