# Supplementary material for: Improvements in Adolescents’ Disordered Eating Behaviors in a Collaborative Care Digital Mental Health Intervention: Retrospective Observational Study
Source: JMIR Form Res. 2024 Jan 31;8:e54253. doi: 10.2196/54253 (PMC10867747; doi:10.2196/54253)
Supplement: Multimedia Appendix 2 [file formative_v8i1e54253_app2.docx]

**Multimedia appendix 2**

To determine which items drove elevated SCOFF scores for members with a SCOFF score of 2 at baseline (the minimum elevated SCOFF score), responses to each item at baseline were assessed for only this group; Table S1. For members with a SCOFF score of 2, 87.0% (n = 87) responded “yes” to the control item (item 2) and 52.0% (n = 52) responded “yes” to the believing you are fat item (item 4). Thus, these two items largely drove inclusion in the elevated SCOFF group. Few members responded “yes” to the making yourself sick item (item 1; 11.0%, n = 11) and the loss of one stone item (item 3; 11.0%, n = 11). Specifically, only 6.5% of all elevated SCOFF members (n = 169) were included due to a “yes” response to one of these two items, indicating that inclusion in the elevated SCOFF score group was not largely driven by responses to these items.

In follow-up analyses, all analyses of change in SCOFF score for those with elevated SCOFF scores were repeated with the response to item 3 excluded from calculation of total SCOFF score. The rates of change, as well as overall change in SCOFF score are reported in Table S2. As with the main analyses, SCOFF scores decreased significantly from baseline to the last assessment when item 3 was removed (*Z* = -5.68, *P* < .001). In the follow-up linear mixed-effects model, SCOFF scores decreased over months in care (F_1,216_ = 55.54, P < .001). Further, elevated anxiety and depressive symptoms predicted higher SCOFF scores (Anxiety: F_1,93_ = 8.29, P = .010; Depressive: F_1,93_ = 4.63, P = .045). The main effect of participation in therapy was not statistically significant (F_1,93_ = 2.84, P = 0.95). The pattern of these follow-up results followed the same as the pattern of results observed in the main analysis, indicating that changes in SCOFF score were not driven by responses to item 3.

**Table S1.** Response rates to each SCOFF item at baseline for members with a baseline SCOFF score of 2.

| **SCOFF question** | **SCOFF score = 2**  **% (n = 100)** |
| --- | --- |
| Do you make yourself **S**ick because you feel uncomfortably full? | 11.0% (n = 11) |
| Do you worry that you have lost **C**ontrol over how much you eat? | 87.0% (n = 87) |
| Have you recently lost more than **O**ne stone (14 lb) in a 3-month period? | 11.0% (n = 11) |
| Do you believe yourself to be **F**at when others say you are too thin? | 52.0% (n = 52) |
| Would you say that **F**ood dominates your life? | 39.0% (n = 39) |

**Table S2.** Change in SCOFF score from baseline to last assessment for members with elevated SCOFF scores after excluding item 3. Baseline and last assessment SCOFF scores, the change in score, and rates of score decrease and increase, are reported.

|  | Elevated SCOFF score  (n = 105) |
| --- | --- |
| SCOFF score: Median (IQR)  Baseline  Last  Change | 2 (1)  2 (2)  -1 (2) |
| Change in score: % (n)  Decrease  Increase | 58.1% (n = 61)  16.2% (n = 17) |
